# Constructs Influencing Patient Perceptions of Use of AI in Medical Imaging Analysis: Systematic Review

**DOI:** 10.2196/92969

**Published:** 2026-06-01

**Authors:** Preksha Machaiya Kuppanda, Monika Janda, Liam J Caffery

**Affiliations:** 1 Frazer Institute Faculty of Health, Medicine and Behavioural Sciences The University of Queensland Brisbane, Queensland Australia; 2 Centre for Online Health The University of Queensland Brisbane, Queensland Australia

**Keywords:** artificial intelligence, patient-centered care, medical imaging, patient engagement, health technology

## Abstract

**Background:**

The use of artificial intelligence (AI) in medical imaging has been growing exponentially. Understanding patient perceptions and factors influencing their views of AI is critical to develop adequate strategies to support implementation and acceptance.

**Objective:**

This study aims to investigate the constructs that influence patients’ perceptions and acceptance of AI’s use in the analysis of their medical images to support screening and diagnosis.

**Methods:**

A systematic review was conducted to meet the research objective. Relevant articles were found by searching 5 databases. Data were extracted using an iteratively refined framework and synthesized narratively due to heterogeneity in study designs, populations, health care contexts, and outcomes.

**Results:**

A total of 59 relevant studies were included in the review. Patient acceptance of AI in medical image analysis emerged from multiple interacting factors. The most consistently reported determinant in 48 studies was that AI implementation should prioritize human-in-the-loop models, positioning AI as supportive tools, working in conjunction with health care providers rather than as an autonomous decision-maker. Other factors identified were performance of the AI, clarity of accountability, trust, and ethical factors. Patients’ individual characteristics such as demographics and health history were also noted to influence acceptance indirectly. The review findings were used to draft a conceptual model to draw attention to the complex relationship among the identified factors.

**Conclusions:**

This review informed the development of a conceptual model illustrating the complex and interactive factors shaping patient acceptance of AI in medical imaging, which can be tested prospectively in future studies. Our results highlight that patients’ likelihood of accepting AI cannot be attributed to a few factors. Instead, promoting acceptance will require a holistic approach where multiple factors are considered simultaneously and adapted for each use case.

## Introduction

The use of artificial intelligence (AI) to support medical imaging analysis has been growing exponentially [1,2]. AI tools are increasingly used for a variety of tasks, ranging from automated image acquisition, sophisticated image reconstruction, classification, and diagnosis [3]. Initially, AI tools were primarily used for tasks like image segmentation or abnormality detection, whereas recent applications have advanced to deep learning–driven complex medical image classification based on large datasets, with minimal human involvement [4]. For example, AI applications have shown promising results and capabilities in the early detection of lung [5], skin [6], and breast cancers [7] from various image types such as computed tomography scans, dermoscopic (magnified) skin images, and mammography scans. In experimental studies, AI’s performance has been comparable or surpassed human readers, including experienced clinicians [8-10]. In the future, AI is expected to contribute widely to the imaging process, including image analysis and interpretation, and reduce burden on health care professionals, ultimately contributing to improved patient outcomes [1,11,12].

Although the potential benefits of AI in medical image analysis are widely recognized and its implementation is proceeding rapidly, its application in health care remains largely confined to experimental and research settings, with limited integration into clinical practice in some medical imaging applications [13]. Lack of confidence and skepticism among health care providers and patients toward novel health care technologies have been reported as contributing factors [14,15]. As technology improves, understanding if perceptions and views change is essential to develop adequate strategies to support the implementation and acceptance of AI [16].

Most existing studies and reviews to date have addressed health care professional [17] or multi-stakeholder [18] perceptions and the factors influencing their acceptance of AI in medical image analysis. Research has explored patient perceptions of AI implementation across various medical imaging domains, identifying factors such as trust, human interaction, transparency, accuracy, and individual-perceived benefits and risks associated with AI as key influences [19]. However, despite this progress, there remains a gap in understanding the full range of factors and how they interact to shape patients' perceptions of AI. Recognizing the multitude of factors and how they interact can enable implementation scientists to suggest strategies to make the AI more acceptable to patients. Furthermore, this can guide the development of information leaflets and consent forms as well as implementation strategies that are patient-centered and allow informed consent of AI use across future use cases.

The aim of this review was therefore to identify the factors that impact patients’ views of AI use in medical imaging applications and to understand how frequently they are reported, how these variables interact, or how contextual elements such as patient demographics, health issues, or the role of AI in the health care process might shape perceptions. The literature was searched with the aim to (1) identify the factors influencing patients’ perceptions of AI in medical image analysis and (2) understand how these factors influence patients' acceptance of AI.

## Methods

### Study Design

We undertook a systematic review of the literature. The SPICE [20] (Setting, Population or perspective, Intervention, Comparison, Evaluation) framework was applied to structure the research question and guide the review process, including the development of the search strategy, establishing the inclusion/exclusion criteria, and selection of relevant articles (Table 1). We followed a 2-phase search strategy (Multimedia Appendix 1), which was developed with the assistance of a university librarian. The protocol of this systematic review was registered with PROSPERO (CRD42024571707) [21]. The finding of this review was reported according to the PRISMA (Preferred Reporting Items for Systematic Reviews and Meta-analysis) guidelines [22].

**Table 1:** SPICE (Setting, Population or perspective, Intervention, Comparison, Evaluation) framework.

SPICE framework	Question	Study details
Setting	What is the context for your question?	Medical imaging AI^a^
Population/Perspective	What is the perspective of your stakeholders or future users of the service or product?	Patient perspectives on medical imaging AI
Intervention	What action is being taken?	Health care practitioner using AI in medical image analysis
Comparison	Are there other/alternative actions or outcomes?	Traditional medical image analysis (if applicable)
Evaluation	What is the outcome measure?	Factors influencing patient perceptions of AI in medical image analysis either independently or in combination with one another

^a^AI: artificial intelligence.

### Screening, Selection, Quality Appraisal, and Data Extraction

The final search was conducted in August 2025. Articles from the search results were uploaded to Covidence for title and abstract screening, followed by review of full text articles based on the inclusion criteria (see Table 2). In this review and its inclusion, the term “patient” refers to either participants recruited in a health care setting or individuals from the public who are potential patients or user personas representing patients. The term “AI” refers to both hypothetical and established/applied interventions, incorporated either in the clinic to analyze, interpret, and process medical images and/or support the screening, assessment or diagnosis process. The term “acceptance” refers broadly to patients’ willingness for AI to be used in their medical image analysis, commonly assessed through their expectations, preference, or comfort with AI outputs and AI use.

The screening of titles and abstracts was independently conducted by 2 reviewers (PMK and LJC). In the instance of disagreements between the reviewers, conflicts were resolved by discussion and comparison of the article under question against the predetermined inclusion and exclusion criteria. Full text review, quality appraisal, and data extraction were performed primarily by one reviewer (PMK); a random subset of articles was assessed by a second reviewer (LJC) to ensure accuracy; and any discrepancies in the extracted data were resolved through discussion between the reviewers.

The Joanna Briggs Institute (JBI) critical appraisal checklist [23] was used to assess study quality, with the appropriate tool applied based on each study’s design (analytical cross-sectional/qualitative). For mixed methods studies, the analytical cross-sectional and qualitative checklists were combined to evaluate the relevant components. As each checklist varies in length, we calculated the proportion of “yes” and “no” responses relative to the total number of relevant questions, excluding items not applicable to the study to compare study quality. Quality appraisal findings were used to inform interpretation of the results; therefore, no studies were excluded based on the quality scores alone.

The data extraction was conducted using an iteratively developed extraction framework, where the extraction fields were determined inductively as patterns emerged across the included studies. This approach was selected to accommodate the conceptual and methodological diversity of the literature and to ensure the comprehensive capture of patient-reported factors influencing perceptions and acceptance of AI in medical image analysis. Data extraction included the author, year of publication, study location, study type, health care context, AI context, recruitment details, number of participants, and the outcome measures (ie, factors influencing patient perceptions of use of AI in their medical image analysis). In this review, a factor was defined as any reported patient-related perception, attitude, trust, concern, acceptance, or preferences regarding the use of AI in medical image analysis.

**Table 2:** Inclusion and exclusion criteria.

Category	Inclusion	Exclusion
General	Peer-reviewed, original research articles in English	Reviews, commentaries, non-English language articles
AI^a^ intervention	AI that analyzes medical images for diagnosis or screening (applied AI or hypothetical contexts)	AI for nondiagnostic tasks (eg, automated image capture, radiotherapy treatment planning, surgical automation)
Purpose/use	Used in clinical care and/or by clinicians to support decision making	Patient-facing AI (eg, direct-to-consumer tools, chatbots, mHealth app-based AI)
Patient focus	Examines patient perceptions, attitudes, trust, acceptance, or experience	No patient-reported factors or perceptions. Perceptions of non-patient groups such as health care providers, health care industry representatives, or medical students
Outcomes of interest	Reports factors influencing patient acceptance or experience of AI	Studies that do not identify influencing factors
Setting and context	Imaging in clinical screening/diagnosis settings (eg, radiology, dermatology, ophthalmology, dentistry)	Nonclinical settings or unrelated applications

^a^AI: artificial intelligence.

### Analysis

A narrative synthesis approach was used for data analysis, as the included studies had heterogeneous methods, population groups, health care specialty, and context of AI. Findings were compared across studies and organized into conceptually similar groups, which were summarized and synthesized narratively to address the research question. As no quantitative synthesis of the data was undertaken, the patterns described here should be understood as conceptual.

## Results

### Screening

**Figure 1:**
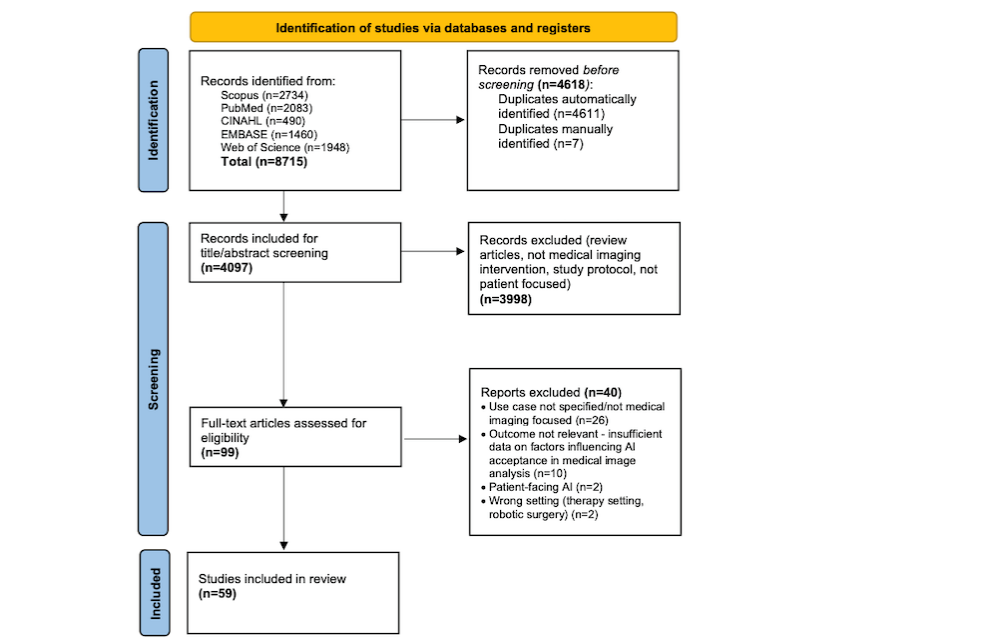
PRISMA (Preferred Reporting Items for Systematic reviews and Meta-Analyses) flowchart of screening and study selection. AI: artificial intelligence.

In this review, 59 studies [15,19,24-80] were included for data extraction, quality appraisal, and analysis. The results of the screening process are detailed in the PRISMA diagram (Figure 1).

### Quality Appraisal

The JBI quality score for the articles ranged from 0.5 to 1 (Table S3 in Multimedia Appendix 1), with higher scores indicating higher quality. Qualitative studies scored highly overall, with lower scores mainly reflecting limited reporting of philosophical positioning. Cross-sectional studies showed greater variability where lower scores were most commonly due to use of nonvalidated questionnaires and limited handling of confounding factors.

### Study Characteristics

The characteristics of the 59 included studies are summarized in Table 3. All included studies were cross-sectional, and 43 studies were quantitative survey–based, with majority (n=20) utilizing de novo, nonvalidated questionnaires, which were informed from literature reviews, existing nonvalidated questionnaires, or expert consultations. Ten included survey studies also adapted the validated “Patients’ Views on the Implementation of AI in Radiology questionnaire” [19]. The qualitative studies mainly used de novo interview guides, with one using prompts adopted from the Theoretical Framework of Acceptability [32] and another mapping its findings to the Theoretical Framework of Acceptability [45]. The included studies were conducted predominantly in Europe (n=34) and focused on radiology-oriented imaging contexts such as mammography (n=13) and general radiology (n=12). The majority of the studies examined hypothetical AI scenarios (n=49).

**Table 3:** Study characteristics (N=59).

Characteristic				Values, n (%)
**Study type**				
	**Quantitative cross-sectional**		43 (73)	
		De novo questionnaire, nonvalidated	20 (34)	
		Adapted from a validated standardized instrument [19]	10 (17)	
		Other validated standardized instruments (eg, Trust in Medical Technology, Client Satisfaction Questionnaire-8 items)	3 (5)	
		De novo questionnaire, partially validated	6 (10)	
		Preference-elicitation instruments (discreet choice/choice-based survey)	4 (7)	
	**Qualitative cross-sectional**		13 (22)	
		Qualitative open-ended survey	1 (2)	
		Focus/dialogue group/engagement workshops	7 (12)	
		Semistructured interviews	5 (8)	
	Mixed methods		3 (5)	
**Study location **				
	Europe		34 (57)	
	Asia		4 (7)	
	Oceania		7 (12)	
	Middle East		3 (5)	
	North America		7 (12)	
	South America		1 (2)	
	Africa		2 (3)	
	Worldwide		1 (2)	
**Medical domain **				
	Mammography/breast imaging		13 (22)	
	Radiology (general, computed tomography scans)		12 (20)	
	Dermatology		9 (15)	
	Ophthalmology		8 (13)	
	Dental		5 (8)	
	Radiology (prostate cancer)		3 (5)	
	Radiology (skeletal)		4 (7)	
	Cardiology		1 (2)	
	Imaging (cervical cancer)		1 (2)	
	Neurosurgery – brain imaging		1 (2)	
	Gastroenterology		1 (2)	
	Dermatology + radiology		1 (2)	
**AI^a^** **application context **				
	Hypothetical AI^b^		49 (83)	
	Applied AI^c^		10 (17)	

^a^AI: artificial intelligence.

^b^Hypothetical AI: studies were classified as hypothetical AI when AI or its output was not shown to patients or did not influence clinical care and perceptions were captured through hypothetical scenarios or questions about AI use.

^c^Applied AI: studies were classified as applied AI when AI results were either shown to patients or used by their health care practitioner to inform clinical decisions, allowing patients to experience its presence and report perceptions in the context of actual AI use.

### Results for Aim (a): Factors Influencing Patients’ Perceptions of AI in Medical Image Analysis

**Figure 2:**
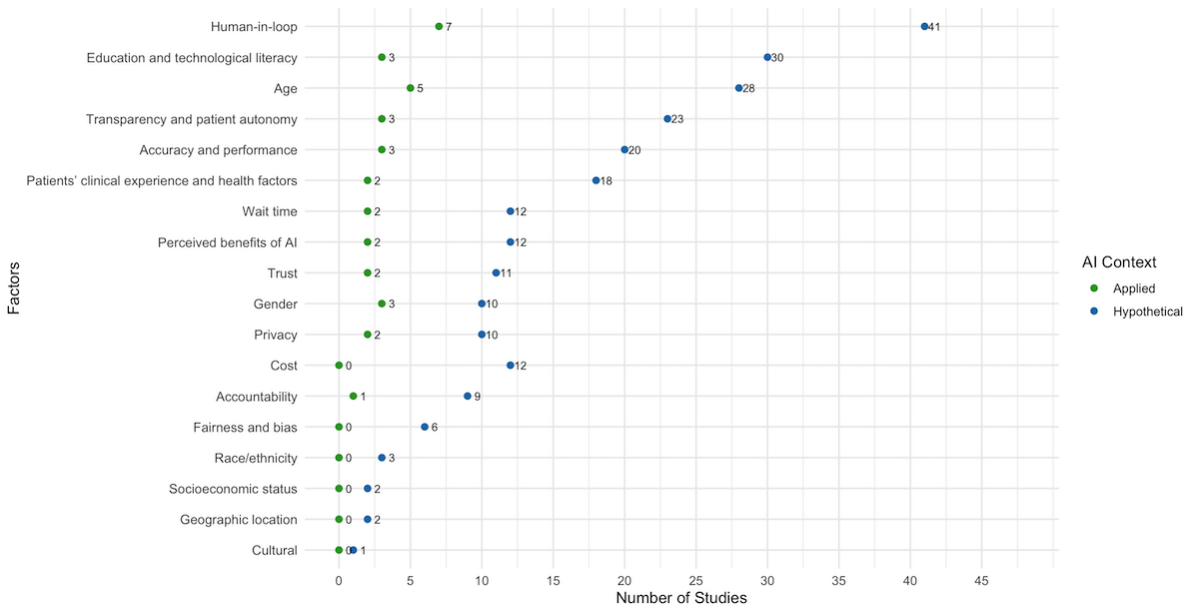
Frequency of the extracted factors across studies with hypothetical and applied artificial intelligence (AI) contexts.

Eighteen factors that influence patient acceptance and perception of AI in medical image analysis were identified and extracted from the 59 included studies (Table S2 in Multimedia Appendix 1). An overview of the factors identified and the frequency of their reporting is depicted in Figure 2.

Human-in-the-loop was identified as the most frequently reported construct across studies and as a key factor influencing and shaping patient acceptance of AI in their medical image analysis. A sensitivity style examination revealed that for both hypothetical and applied AI contexts individually, the top 5 constructs identified were consistent, although their relative ranking varied slightly in the smaller applied AI sample. This, therefore, suggests that perceptions elicited in hypothetical scenarios broadly reflect those observed in applied clinical settings.

### Results for Aim (b): How the Identified Factors Influence Patients' Acceptance of AI

Based on findings from aim (a) and the factors identified, patients’ acceptance of AI in medical image analysis appears to arise through a complex interaction among factors, rather than major effects of individual factors.

**Figure 3:**
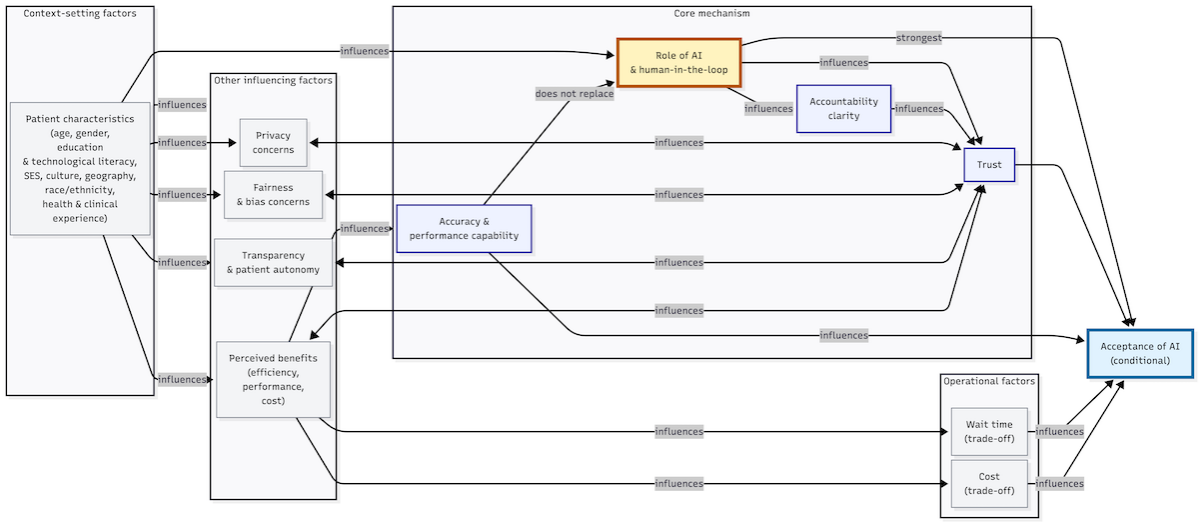
Synthesized conceptual model of factors influencing patients’ acceptance of artificial intelligence (AI) in medical image analysis. SES: socioeconomic status.

Figure 3 is a visual representation of the multifaceted relationships between factors and serves as a conceptual model grounded in associations reported across the included studies. Arrows represent influences identified through narrative synthesis and reflect patterns in participants’ preferences and perceptions rather than empirically tested causal pathways. The model should be interpreted as an author-synthesized framework illustrating how factors discussed across studies may interact in shaping patient acceptance of AI in medical image analysis and viewed alongside the detailed narrative synthesis in the Multimedia Appendix 1 for full context.

Across multiple studies, human-in-the-loop deployment was reported as a central factor associated with higher acceptance of AI. Accountability was frequently discussed alongside human-in-the-loop deployment, reflecting patients’ expectations that clinicians retain responsibility and oversight of AI use. Trust appeared central in how patients evaluated AI, shaping how perceived benefits, risks, and implementation features translated into acceptance. The accuracy of the AI system was commonly reported as a necessary factor for acceptance; however, its impact on acceptance is dependent on human involvement and accountable care models.

Studies reported that the preference and importance placed on the human-in-the-loop model was shaped by patients’ individual characteristics such as demographic factors, health issues and history, and sociodemographic characteristics. These factors serve as contextual factors and influence elements such as patients’ perceived benefits of AI, their privacy, fairness, and bias concerns, and demand for transparency and autonomy, which in turn shapes trust. Operational factors such as cost and wait time were also noted to influence the acceptance of AI, either as perceived benefits or as trade-offs, with some patients willing to compromise on factors such as human involvement, to achieve improvements in operational conditions or to tolerate less favorable operational conditions in return for retaining human involvement.

## Discussion

Patients’ acceptance of AI in medical image analysis was observed to be influenced by several interacting factors. How the AI was applied in the health care setting and human involvement (ie, human-in-the-loop) was most consistently reported to shape acceptance. Hence, high acceptance was reported by studies when AI was positioned as a supportive tool assisting clinicians rather than as functioning autonomously. Other common influential factors included AI accuracy and performance, transparency that AI was used, trust in AI, clarity on who is accountable, and ethical concerns such as privacy and fairness. Sociodemographic characteristics, clinical and health experiences, and operational factors such as waiting time and cost shaped acceptance in more variable and context-dependent ways.

The level of human involvement in how AI is used had the most profound impact on patients' acceptance across studies. These findings are consistent with existing ethical guidelines and empirical studies emphasizing the importance of human oversight and accountability in AI deployment [81-83]. For example, the High-Level Expert Group on Artificial Intelligence listed “human agency and oversight” and “accountability” as a key requirement of a trustworthy AI. Furthermore, Australia’s AI ethics principles state that “People responsible for the different phases of the AI system lifecycle should be identifiable and accountable for the outcomes of the AI systems, and human oversight of AI systems should be enabled.” Furthermore, prior work has shown that acceptance of AI-supported services increases when users are informed that humans remain in control, while fear of human replacement can undermine acceptance [84-88]. This pattern is reflected in many emerging AI applications in medical imaging, where systems are typically designed to support clinician decision-making rather than replace it, such as AI tools developed for ultrasound-based disease classification and magnetic resonance imaging lesion detection [89,90].

AI-based image analysis systems are increasingly capable of high diagnostic performance, reflecting the ongoing emphasis among the emerging AI interventions to enhance diagnostic accuracy [91-93]. However, as identified in this review, although the accuracy of AI was frequently prioritized by patients, it did not override the preference for human involvement in the use of AI. Similarly, the emerging AI systems are typically designed to support clinician decision-making rather than operate autonomously, reflecting that these models account for human-in-the-loop requirement. Furthermore, similar to previous research, this review found that transparency and explainability support acceptance primarily by boosting patients’ trust rather than through inducing a detailed technical understanding of AI among them [94-96].

Studies reported that factors such as privacy and fairness influenced how patients interpret the benefits and risks of AI, thereby shaping their trust and accordingly acted as either facilitators or barriers of acceptance. Concerns related to privacy, fairness, and bias among patients mirror growing evidence that these concerns can undermine trust in AI-enabled health care [88]. Age, gender, and education of patients were the most commonly studied demographic factors; however, correlations with patients' optimism toward AI were not consistent. The inconsistent influence observed in this review aligns with literature on technology acceptance, which also found that demographic characteristics do not directly influence acceptance. Studies on technology acceptance more broadly not specific to AI found that demographic characteristics such as education shape an individual’s technological familiarity, perceived benefits, and expectations of the intervention, which ultimately influences their acceptance [97]. Despite this, acceptance can be high or low within each demographic subgroup and can change over time, as more information about a new technology becomes available and therefore needs to be assessed in future studies.

Patients’ individual characteristics like their medical history, experience with clinical contexts, and personal psychological factors were noted to act as context-setting factors, influencing expectations and sensitivities around AI. Operational factors such as waiting times and costs were identified as trade-offs, with patients potentially willing to trade-off some of their health care professionals’ involvement and accept autonomous use of AI in their screening or diagnosis for shorter wait times and reduced costs, or vice versa.

This study has several limitations. First, the conceptual model is grounded in data extracted from the included studies and informed by the authors’ interpretation; however, given the complexity of the relationships identified, not all potential interactions could be exhaustively represented. Second, the heterogeneous nature of the included studies and the use of varying survey tools limited direct comparison and quantitative synthesis. Third, certain factors such as cultural influence, socioeconomic status, and race or ethnicity were underreported across studies. This may limit the depth of conclusions that can be drawn in these areas and bias the findings toward populations and health care settings most commonly represented in the literature. This, therefore, may limit the generalizability of conclusions to more diverse contexts. Fourth, variations in terminology and conceptualization of “acceptance” across studies may affect comparability. Fifth, while the search terms and search strategy was developed with adequate guidance to be as exhaustive as possible, there is potential that some relevant publications may have not made into the search results due to variations in the keywords or controlled vocabulary used. Finally, our review focused on peer-reviewed sources reporting original research, including letters or short reports with sufficient methodological detail. Therefore, the findings may emphasize concerns that are more likely to be published, such as privacy, fairness, or specific medical contexts, and may underrepresent perspectives reported in conference proceedings, regional reports, or grey literature. This limitation should be considered when interpreting the results and designing future studies.

Overall, this review suggests that successful implementation of AI in medical image analysis should prioritize human-in-the-loop designs. Policymakers and health care organizations should focus on building governance frameworks that strengthen trust, address privacy and fairness concerns, and ensure that responsibility for AI-assisted decisions remains clearly assigned to human professionals. Furthermore, considering the complex relationship among these influencing factors, with one impacting the other in most instances, patients’ choice of accepting AI cannot be attributed to one or a few factors. Instead, promoting acceptance will require a holistic approach where all influencing factors are considered on a case-by-case basis. 

Having a standard set of constructs and variables to assess patients’ acceptance may be beneficial and enable the development of a structured approach to enhance patient acceptance of AI. The influencing factors identified in this review can form the basis for the development of standard measurement constructs to understand patients' acceptability of AI in medical image analysis for screening and diagnosis. These constructs can be further explored and substantiated by discussions and interviews with patients.

It is important to note that the relationships represented in Figure 3 should not be interpreted as causal pathways. Most included studies were cross-sectional surveys that assessed reported preferences or perceptions, often using hypothetical scenarios. As such, the model represents an interpretive synthesis of reported associations and patterns in participants’ reasoning rather than empirically tested mechanisms. The framework is therefore intended to guide future research that could prospectively examine these relationships.

## Appendix

Multimedia Appendix 1 PRISMA checklist.

## Abbreviations

AI: artificial intelligence
JBI: Joanna Briggs Institute
PRISMA: Preferred Reporting Items for Systematic Reviews and Meta-analysis
SPICE: Setting, Population or perspective, Intervention, Comparison, Evaluation

## Data Availability

This systematic review was a secondary study; therefore, data availability is not applicable to this article. Details of the search strategy are included in the Multimedia Appendix 1.
