# Supplementary material for: Constructs Influencing Patient Perceptions of Use of AI in Medical Imaging Analysis: Systematic Review
Source: Interact J Med Res. 2026 Jun 1;15:e92969. doi: 10.2196/92969 (PMC13270170; doi:10.2196/92969)
Supplement: Multimedia Appendix 1 [file ijmr_v15i1e92969_app1.docx]

Multimedia Appendix

Table of Contents

[PRISMA checklist 2](#_Toc230182559)

[Search strategy 5](#_Toc230182560)

[Quality appraisal 8](#_Toc230182561)

[Data extraction and sensitivity analysis 8](#_Toc230182562)

[Factors extracted across the included studies 10](#_Toc230182563)

[Results for aim (a): factors influencing patients’ perceptions of AI in medical image analysis 13](#_Toc230182564)

[Sociodemographic factors 13](#_Toc230182565)

[Clinical experience and health factors 15](#_Toc230182566)

[Psychosocial and ethical concerns 16](#_Toc230182567)

[Attributes of the AI-enabled service 17](#_Toc230182568)

[Operational factors of the health care system 20](#_Toc230182569)

[Summary characteristics of included studies 22](#_Toc230182570)

[References 25](#_Toc230182571)

PRISMA checklist ^[1]^

| **Section and Topic** | **Item #** | **Checklist item** | **Location where item is reported** |
| --- | --- | --- | --- |
| **TITLE** | | |  |
| Title | 1 | Identify the report as a systematic review. | Title (Manuscript) |
| **ABSTRACT** | | |  |
| Abstract | 2 | See the PRISMA 2020 for Abstracts checklist. | Abstract (Manuscript) |
| **INTRODUCTION** | | |  |
| Rationale | 3 | Describe the rationale for the review in the context of existing knowledge. | Introduction, para 2, 3 (Manuscript) |
| Objectives | 4 | Provide an explicit statement of the objective(s) or question(s) the review addresses. | Introduction, para 4 aims (Manuscript) |
| **METHODS** | | |  |
| Eligibility criteria | 5 | Specify the inclusion and exclusion criteria for the review and how studies were grouped for the syntheses. | Methods – Table 1, Table 2 (Manuscript) |
| Information sources | 6 | Specify all databases, registers, websites, organisations, reference lists and other sources searched or consulted to identify studies. Specify the date when each source was last searched or consulted. | Fig 1 (Manuscript) |
| Search strategy | 7 | Present the full search strategies for all databases, registers and websites, including any filters and limits used. | Search strategy (Multimedia Appendix) |
| Selection process | 8 | Specify the methods used to decide whether a study met the inclusion criteria of the review, including how many reviewers screened each record and each report retrieved, whether they worked independently, and if applicable, details of automation tools used in the process. | Methods Section - Screening, selection, quality appraisal, and data extraction, Table 2 – inclusion and exclusion criteria (Manuscript) |
| Data collection process | 9 | Specify the methods used to collect data from reports, including how many reviewers collected data from each report, whether they worked independently, any processes for obtaining or confirming data from study investigators, and if applicable, details of automation tools used in the process. | Methods Section - Screening, selection, quality appraisal, and data extraction, para 2 (Manuscript) |
| Data items | 10a | List and define all outcomes for which data were sought. Specify whether all results that were compatible with each outcome domain in each study were sought (e.g. for all measures, time points, analyses), and if not, the methods used to decide which results to collect. | Methods Section - Screening, selection, quality appraisal, and data extraction, para 4 (Manuscript) |
|  | 10b | List and define all other variables for which data were sought (e.g. participant and intervention characteristics, funding sources). Describe any assumptions made about any missing or unclear information. | Methods Section - Screening, selection, quality appraisal, and data extraction (Manuscript) |
| Study risk of bias assessment | 11 | Specify the methods used to assess risk of bias in the included studies, including details of the tool(s) used, how many reviewers assessed each study and whether they worked independently, and if applicable, details of automation tools used in the process. | Section - Screening, selection, quality appraisal, and data extraction  , Results Section – Quality Appraisal (Manuscript) |
| Effect measures | 12 | Specify for each outcome the effect measure(s) (e.g. risk ratio, mean difference) used in the synthesis or presentation of results. | Formal quantitative effect measures were not synthesised due to heterogeneity; results were compared narratively |
| Synthesis methods | 13a | Describe the processes used to decide which studies were eligible for each synthesis (e.g. tabulating the study intervention characteristics and comparing against the planned groups for each synthesis (item #5)). | All included studies were considered together within a single narrative synthesis; no formal grouping of studies was undertaken beyond organising extracted findings into conceptually similar categories |
|  | 13b | Describe any methods required to prepare the data for presentation or synthesis, such as handling of missing summary statistics, or data conversions. | Methods section - Screening, selection, quality appraisal, and data extraction - Conceptual data preparation for narrative synthesis; Iterative extraction framework and inductive refinement; no numerical transformation required (Manuscript) |
|  | 13c | Describe any methods used to tabulate or visually display results of individual studies and syntheses. | Tabe S1 ((Multimedia Appendix), Table 3 (Manuscript) |
|  | 13d | Describe any methods used to synthesize results and provide a rationale for the choice(s). If meta-analysis was performed, describe the model(s), method(s) to identify the presence and extent of statistical heterogeneity, and software package(s) used. | Methods Section- Screening, selection, quality appraisal, and data extraction - Synthesis of results was conducted using a narrative approach. This approach was selected because the included studies were heterogeneous, which precluded quantitative pooling of results. (Manuscript) |
|  | 13e | Describe any methods used to explore possible causes of heterogeneity among study results (e.g. subgroup analysis, meta-regression). | No formal methods were used to explore potential sources of heterogeneity among study results. Given the narrative nature of the synthesis and the focus on descriptive integration of findings, subgroup analyses or other heterogeneity investigations were not undertaken. |
|  | 13f | Describe any sensitivity analyses conducted to assess robustness of the synthesized results. | NA- Sensitivity analyses were not conducted because the review used a narrative synthesis and did not include a meta-analysis |
| Reporting bias assessment | 14 | Describe any methods used to assess risk of bias due to missing results in a synthesis (arising from reporting biases). | Not done |
| Certainty assessment | 15 | Describe any methods used to assess certainty (or confidence) in the body of evidence for an outcome. | Not done |
| **RESULTS** | | |  |
| Study selection | 16a | Describe the results of the search and selection process, from the number of records identified in the search to the number of studies included in the review, ideally using a flow diagram. | Figure 1 (Manuscript) |
|  | 16b | Cite studies that might appear to meet the inclusion criteria, but which were excluded, and explain why they were excluded. | NA |
| Study characteristics | 17 | Cite each included study and present its characteristics. | Table S2, S3 (Multimedia Appendix), Table 3 (Manuscript) |
| Risk of bias in studies | 18 | Present assessments of risk of bias for each included study. | Table S1 (Multimedia Appendix), JBI quality score |
| Results of individual studies | 19 | For all outcomes, present, for each study: (a) summary statistics for each group (where appropriate) and (b) an effect estimate and its precision (e.g. confidence/credible interval), ideally using structured tables or plots. | Factors extracted- Table S1, S2 (Multimedia Appendix). Narrative synthesis of results; no meta-analysis conducted |
| Results of syntheses | 20a | For each synthesis, briefly summarise the characteristics and risk of bias among contributing studies. | Results Section – Quality Appraisal (Manuscript) |
|  | 20b | Present results of all statistical syntheses conducted. If meta-analysis was done, present for each the summary estimate and its precision (e.g. confidence/credible interval) and measures of statistical heterogeneity. If comparing groups, describe the direction of the effect. | Narrative synthesis of results; no meta-analysis conducted |
|  | 20c | Present results of all investigations of possible causes of heterogeneity among study results. | No investigations were conducted to examine potential causes of heterogeneity among study results |
|  | 20d | Present results of all sensitivity analyses conducted to assess the robustness of the synthesized results. | Data extraction and sensitivity analysis (Multimedia Appendix) Analysis was conducted to examine whether the top 5 factors extracted persisted when the JBI quality score of the studies varied (high, medium, and low) and across both application contexts of AI (hypothetical and applied). |
| Reporting biases | 21 | Present assessments of risk of bias due to missing results (arising from reporting biases) for each synthesis assessed. | No reporting bias assessment performed |
| Certainty of evidence | 22 | Present assessments of certainty (or confidence) in the body of evidence for each outcome assessed. | No certainty assessment conducted |
| **DISCUSSION** | | |  |
| Discussion | 23a | Provide a general interpretation of the results in the context of other evidence. | Discussion, para 1 |
|  | 23b | Discuss any limitations of the evidence included in the review. | Discussion, para 6 |
|  | 23c | Discuss any limitations of the review processes used. | Discussion, para 6 |
|  | 23d | Discuss implications of the results for practice, policy, and future research. | Discussion, para 7, 8 |
| **OTHER INFORMATION** | | |  |
| Registration and protocol | 24a | Provide registration information for the review, including register name and registration number, or state that the review was not registered. | Registered, Methods, Study Design - para 1 (Manuscript) |
|  | 24b | Indicate where the review protocol can be accessed, or state that a protocol was not prepared. | Not prepared |
|  | 24c | Describe and explain any amendments to information provided at registration or in the protocol. | NA |
| Support | 25 | Describe sources of financial or non-financial support for the review, and the role of the funders or sponsors in the review. | Funding (Manuscript) |
| Competing interests | 26 | Declare any competing interests of review authors. | Included |
| Availability of data, code and other materials | 27 | Report which of the following are publicly available and where they can be found: template data collection forms; data extracted from included studies; data used for all analyses; analytic code; any other materials used in the review. | NA |

# Search strategy

To identify relevant studies, 5 databases were searched: PubMed, CINAHL, EMBASE, SCOPUS, and Web of Science. We followed a two-phase search strategy, with an initial scoping search conducted to identify key studies and refine our search terms and keywords. This was followed by a Boolean search comprising relevant keywords (e.g. patient perceptions, medical imaging, artificial intelligence), synonyms, and controlled vocabulary (e.g. medical subject headings [MeSH], Emtree). The search strategy was developed with the assistance of a university librarian. The primary search was conducted in July 2024 and updated later in August 2025.

The database searches included articles, conference papers, editorials, letters, and short surveys. During screening, only peer-reviewed journal articles reporting original research in English were included. Letters, short surveys, or brief reports were included only if they presented original data on patients’ perceptions of AI in health care with clearly described methodology and outcomes. Conference papers, editorials, and other non-peer-reviewed literature were excluded. Grey literature (e.g., theses, technical reports) was excluded by design.


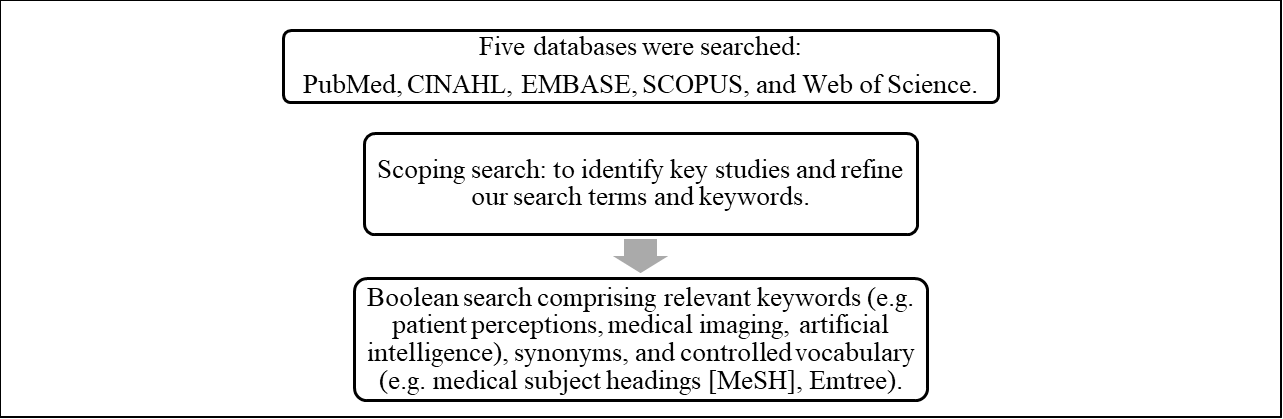


**PubMed – Complete search done 1900/01/01 - 2025-08-28 – 2083 results (English only)**

**(**("patient*"[tiab] OR “public”[tiab] OR “population*”[tiab] OR “women*”[tiab] OR “men”[tiab] OR “mens”[tiab]) AND ("attitude*"[tiab] OR "perspective*"[tiab] OR "perception*"[tiab] OR "patient* views"[tiab] OR "preference*"[tiab] OR "trust"[tiab] OR “acceptance”[tiab] OR “acceptability”[tiab] OR “satisfaction”[tiab] OR “judgement*”[tiab])**)**

**AND**

("Diagnostic Imaging"[Majr] OR "Radiology"[Majr] OR "radiography"[tiab] OR "radiology"[tiab] OR "medical imaging"[tiab] OR "imaging"[tiab] OR "scan*"[tiab] OR “image analysis”[tiab] OR “screening”[tiab] OR “diag*”[tiab])

**AND**

("Artificial Intelligence"[Majr] OR "artificial intelligence"[ti] OR "AI"[ti] OR "machine learning"[ti] OR "deep learning"[ti] OR "machine intelligence"[ti] OR “Convolutional Neural Networks” [ti] OR “CNN”[ti] OR "computer assisted"[ti] OR "computer-assisted"[ti] OR “computer aided”[ti] OR “computer-aided”[ti] OR "automated"[ti])

**Filters**: remove review and systematic reviews

**Embase (Elsevier) – Complete search done 1900/01/01 - 2025-08-28 – 1460 results (English only)**

**(**("patient*":ti,ab  OR “public”:ti,ab  OR “population*”:ti,ab OR “women*”:ti,ab OR “men”:ti,ab OR “mens”:ti,ab) AND ("attitude*":ti,ab OR "perspective*":ti,ab OR "perception*":ti,ab OR "patient* views":ti,ab  OR "preference*":ti,ab OR "trust":ti,ab  OR “acceptance”:ti,ab  OR “satisfaction”:ti,ab OR “judgement*”:ti,ab)**)**

**AND**

('diagnostic imaging'/exp/mj OR 'diagnostic imaging equipment'/exp/mj OR 'radiology'/exp/mj OR "radiography":ti,ab  OR "radiology":ti,ab  OR "medical imaging":ti,ab  OR "imaging":ti,ab  OR "scan*":ti,ab  OR “image analysis”:ti,ab OR “screening”:ti,ab OR “diag*”:ti,ab)

**AND**

('artificial intelligence'/exp/mj OR "artificial intelligence":ti OR "AI":ti OR "machine learning":ti OR "deep learning":ti OR "machine intelligence":ti OR “Convolutional Neural Networks”:ti OR “CNN”:ti OR "computer assisted":ti OR "computer-assisted":ti OR “computer aided”:ti OR “computer-aided”:ti OR "automated":ti)

**AND**('article'/it OR 'conference paper'/it OR 'editorial'/it OR 'short survey'/it)

**CINAHL Complete (EBSCOhost) – Complete search done 1900/01/01 - 2025-08-28 – 490 results (English only)**

**(**(TI("patient*" OR "public" OR "population*" OR “women*”OR “men” OR “mens”) OR AB("patient*" OR "public" OR "population*" OR “women*”OR “men” OR “mens”)) AND (TI("attitude*" OR "perspective*" OR  "perception*" OR "patient* views" OR "preference*" OR "trust" OR “acceptance” OR “satisfaction” OR “judgement*”) OR AB(“attitude*" OR "perspective*" OR  "perception*" OR "patient* views" OR "preference*" OR "trust" OR “acceptance” OR “satisfaction” OR “judgement*”))**)**

**AND**

(MM "Diagnostic Imaging+" OR TI("radiography" OR "radiology" OR "medical imaging" OR "imaging" OR "scan*" OR “image analysis” OR “screening” OR “diag*”) OR AB("radiography" OR "radiology" OR "medical imaging" OR "imaging" OR "scan*" OR “image analysis” OR “screening” OR “diag*”))

**AND**

(MM "Artificial Intelligence+" OR TI("artificial intelligence" OR "AI" OR "machine learning" OR "deep learning" OR "machine intelligence" OR "computer assisted" OR "computer-assisted" OR “computer aided” OR “computer-aided” OR "automated"))

Filter: Academic Journals

**Scopus, Advanced Search (Elsevier) –  Complete search done 1900/01/01 - 2025-08-28 – 2734 results (English only)**

**(**(TITLE-ABS("patient*" OR "public" OR "population*" OR “women*”OR “men” OR “mens”)) AND (TITLE-ABS("attitude*" OR "perspective*" OR  "perception*" OR "patient* views" OR "preference*" OR "trust" OR “acceptance” OR “satisfaction” OR “judgement*”))**)**

**AND**

(TITLE-ABS(“radiography" OR "radiology" OR "medical imaging" OR "imaging" OR "scan*" OR “image analysis” OR “screening” OR “diag*”))

**AND**

(TITLE("artificial intelligence" OR "AI" OR "machine learning" OR "deep learning" OR "machine intelligence" OR "computer assisted" OR "computer-assisted" OR “computer aided” OR “computer-aided” OR "automated"))

**AND**

( LIMIT-TO ( DOCTYPE , "ar" ) OR LIMIT-TO ( DOCTYPE , "cp" ) OR LIMIT-TO ( DOCTYPE , "sh" ) OR LIMIT-TO ( DOCTYPE , "ed" ) )

**Web of Science Core Collection All Editions, Advanced Search (Clarivate) – Complete search done 1900/01/01 - 2025-08-28 – 1948 results (English only)**

**(**(TI=("patient*" OR "public" OR "population*" OR “women*”OR “men” OR “mens”) OR AB=("patient*" OR "public" OR "population*" OR “women*”OR “men” OR “mens”)) AND (TI=("attitude*" OR "perspective*" OR  "perception*" OR "patient* views" OR "preference*" OR "trust" OR “acceptance” OR “satisfaction” OR “judgement*”) OR AB=("attitude*" OR "perspective*" OR  "perception*" OR "patient* views" OR "preference*" OR "trust" OR “acceptance” OR “satisfaction” OR “judgement*”))**)**

**AND**

(TI=(“radiography" OR "radiology" OR "medical imaging" OR "imaging" OR "scan*" OR “image analysis” OR “screening” OR “diag*”) OR AB=(“radiography" OR "radiology" OR "medical imaging" OR "imaging" OR "scan*" OR “image analysis” OR “screening” OR “diag*”))

**AND**

(TI=("artificial intelligence" OR "AI" OR "machine learning" OR "deep learning" OR "machine intelligence" OR "computer assisted" OR "computer-assisted" OR “computer aided” OR “computer-aided” OR "automated"))

**AND**

Article or Proceeding Paper or Editorial Material or Early Access (Document Types)

# Quality appraisal

The Joanna Briggs Institute (JBI) critical appraisal checklist [2] was used to assess study quality and the quality cutoffs were set as follows: a high-quality score (0.80 or more), a medium-quality score (0.60-0.79), and low-quality score (<0.59). The JBI quality score for the articles ranged from 0.5 to 1, with forty studies receiving a high-quality score, fifteen a medium-quality score, and four a low-quality score.

# Data extraction and sensitivity analysis

The frequency of factors extracted across the included studies, the two AI contexts, and the three-study quality have been depicted in Table S1 and Table S2. When extracting factors**,** each study contributed only once to a given factor.

Analysis was conducted to examine whether the top 5 factors extracted, including role of AI and human-in-the-loop, age, education, transparency and patient autonomy, and accuracy and performance of AI persisted when the JBI quality score of the studies varied (high, medium, and low) and across both application contexts of AI (hypothetical and applied).

A Fisher–Freeman–Halton exact test using Monte Carlo simulation indicated no significant difference in the frequency of factors extracted between hypothetical and applied AI contexts (p = 0.953). Similarly, the prevalence of factors extracted did not differ significantly across studies with high, medium, or low JBI quality scores (p = 0.983). This suggests that the most frequently coded themes were consistently identified regardless of AI type or study quality.

Sensitivity style exploration further confirmed the robustness of these findings. Factors were considered only within hypothetical AI studies and only within high-quality studies, and it was noted that the top five most frequently extracted factors remained unchanged (see Table S1). These results indicate that the key conclusions of the review are robust to study quality and AI context.

Table S1: Frequency of Extracted Themes Across AI Context and Study Quality

| Factor | Total count | Hypothetical AI | Applied AI | High quality JBI score | Medium quality JBI score | Low quality JBI score |
| --- | --- | --- | --- | --- | --- | --- |
| No. of articles | 59 | 48 | 11 | 40 | 15 | 4 |
| Role of AI and human-in-the-loop | 48 | 41 | 7 | 32 | 14 | 2 |
| Age | 33 | 28 | 5 | 22 | 9 | 2 |
| Education and technological literacy | 33 | 30 | 3 | 23 | 8 | 2 |
| Transparency and patient autonomy | 26 | 23 | 3 | 19 | 6 | 1 |
| Accuracy and performance | 23 | 20 | 3 | 18 | 5 | 0 |
| Patients’ clinical experience and health factors | 20 | 18 | 2 | 12 | 7 | 1 |
| Perceived benefits of AI | 14 | 12 | 2 | 12 | 2 | 0 |
| Wait time | 14 | 12 | 2 | 6 | 8 | 0 |
| Gender | 13 | 10 | 3 | 10 | 1 | 2 |
| Trust | 13 | 11 | 2 | 11 | 2 | 0 |
| Privacy | 12 | 10 | 2 | 8 | 4 | 0 |
| Cost | 12 | 12 | 0 | 10 | 2 | 0 |
| Accountability | 10 | 9 | 1 | 7 | 2 | 0 |
| Fairness and bias | 6 | 6 | 0 | 5 | 1 | 0 |
| Race/ethnicity | 3 | 3 | 0 | 2 | 1 | 0 |
| Socioeconomic status | 2 | 2 | 0 | 2 | 0 | 0 |
| Geographic location | 2 | 2 | 0 | 2 | 0 | 0 |
| Cultural | 1 | 1 | 0 | 0 | 1 | 0 |

# Factors extracted across the included studies

**Table S2:** Factors extracted across the included studies

| **Extracted factors**  **Study** | Role of AI and human-in-the-loop | Age | Education | Transparency and patient autonomy | Accuracy and performance | Clinical experience and health factors | perceived benefits | Wait time | Gender | Trust | Privacy | Cost | Accountability | Fairness and bias | Race/ethnicity | SES | Geographic location | Cultural |
| --- | --- | --- | --- | --- | --- | --- | --- | --- | --- | --- | --- | --- | --- | --- | --- | --- | --- | --- |
| Adams 2020 [3] | X |  |  | X | X |  |  | X |  | X | X |  |  |  |  |  |  | X |
| Agarwal 2025 [4] | X |  | X |  | X | X |  |  | X |  |  |  |  |  | X |  |  |  |
| Arora 2025 [5] | X | X | X |  |  |  |  |  |  |  |  | X |  |  |  |  | X |  |
| Ayad 2023 [6] |  | X |  | X | X |  |  |  |  |  |  | X |  |  |  |  |  |  |
| Baghdadi 2024 [7] | X | X | X | X |  | X |  |  | X |  |  |  |  |  |  |  |  |  |
| Bahadir 2025 [8] |  | X | X |  |  |  |  |  |  |  |  |  |  |  |  |  |  |  |
| Bahakeem 2023 [9] | X | X | X | X |  | X |  | X |  |  |  |  |  |  |  |  |  |  |
| Baillie 2025 [10] | X |  |  | X | X |  | X | X |  | X | X |  |  |  |  |  |  |  |
| Carter 2023 [11] | X |  | X | X | X |  |  | X |  | X | X | X | X |  |  |  |  |  |
| Catalina 2023 [12] | X | X | X |  |  |  | X |  | X |  |  |  | X |  |  |  |  |  |
| Clements 2022 [13] | X |  | X |  | X |  |  |  | X |  |  | X | X |  |  |  |  |  |
| Dontchos 2025 [14] |  | X | X |  |  |  |  |  |  | X | X |  |  | X | X |  |  |  |
| El-Sayed 2025 [15] | X |  |  | X |  |  | X |  |  | X | X | X |  |  |  |  |  |  |
| Fink 2018 [16] | X | X |  | X |  | X |  |  | X |  |  |  |  |  |  |  |  |  |
| Foresman 2025 [17] | X |  |  | X | X |  |  |  |  |  |  |  |  | X |  |  |  |  |
| Fransen 2025 [18] | X | X | X |  | X |  |  |  |  |  |  |  | X |  |  |  |  |  |
| Frühauf 2012 [19] | X | X |  |  |  |  |  |  | X |  |  |  |  |  |  |  |  |  |
| Gatting 2024 [20] | X |  |  | X | X |  | X |  |  |  | X |  |  | X |  |  |  |  |
| Goessinger 2024 [21] | X | X | X |  | X |  | X |  |  |  |  |  |  |  |  |  |  |  |
| Haan 2019 [22] | X |  | X | X | X |  | X | X |  | X |  |  | X |  |  |  |  |  |
| Haggenmüller 2024 [23] |  |  |  | X | X | X |  |  |  |  |  |  |  |  |  |  |  |  |
| Holen 2024 [24] | X | X | X | X |  | X |  |  |  |  |  |  |  |  |  |  |  |  |
| Ibba 2023 [25] | X | X | X |  |  |  |  |  |  |  |  |  |  |  |  |  |  |  |
| Jagemann 2024 [26] | X | X | X |  |  |  |  | X | X |  | X | X |  |  |  |  |  |  |
| Johansson 2024 [27] | X |  |  |  | X |  |  |  |  | X | X |  | X |  |  |  |  |  |
| Jonmarker 2019 [28] | X | X | X |  |  |  |  | X |  |  |  |  |  |  |  |  |  |  |
| Jutzi 2020 [29] | X | X | X | X | X | X |  | X |  |  | X | X |  |  |  |  |  |  |
| Kawsar 2023 [30] | X |  |  |  |  | X |  | X |  |  |  |  |  |  |  |  |  |  |
| Keel 2018 [31] |  |  |  |  |  |  |  | X |  |  |  |  |  |  |  |  |  |  |
| Kosan 2022 [32] | X | X | X | X |  |  |  |  |  |  |  |  |  |  |  |  |  |  |
| Lee 2024 [33] | X |  |  |  | X | X |  | X |  |  |  |  |  |  |  |  |  |  |
| Lennox-Chhugani 2021 [34] | X | X |  |  | X |  |  |  |  | X |  |  |  |  |  |  |  |  |
| Lim 2022 [35] | X | X |  |  |  | X |  |  |  |  |  |  |  |  |  |  |  |  |
| Lyso 2024 [36] | X | X |  | X |  | X | X |  |  | X |  |  |  |  |  |  |  |  |
| Malerbi 2024 [37] | X |  | X |  |  |  |  |  |  |  |  |  |  |  |  |  |  |  |
| Manning 2023 [38] | X | X | X | X | X | X | X |  |  | X | X | X |  |  |  |  |  |  |
| McGhee 2025 [39] | X |  |  | X |  |  | X |  |  |  |  | X |  |  |  | X |  |  |
| Nelson 2020 [40] | X |  |  | X | X |  |  | X |  |  |  | X |  |  |  |  |  |  |
| Ongena 2020 [41] | X | X | X | X |  |  |  |  | X |  |  |  |  |  |  |  |  |  |
| Ongena 2021 [42] | X |  | X |  |  |  |  |  |  |  |  |  |  |  |  |  |  |  |
| Ozcan 2025 [43] | X | X | X | X |  | X |  | X |  |  | X |  | X | X | X | X |  |  |
| Palmisciano 2020 [44] | X | X | X | X | X |  |  |  |  |  |  |  |  |  |  |  |  |  |
| Pearce 2025 [45] | X |  |  |  | X | X |  |  |  | X |  |  |  | X |  |  |  |  |
| Pelayo 2023 [46] | X |  |  |  | X |  | X |  |  | X |  |  |  |  |  |  |  |  |
| Pesapane 2023 [47] | X | X | X |  |  | X | X |  |  |  |  |  | X |  |  |  |  |  |
| Popic 2025 [48] | X |  |  |  | X |  |  |  |  |  |  |  |  |  |  |  |  |  |
| Rodler 2024 [49] | X | X | X |  |  | X |  |  |  |  |  |  |  |  |  |  |  |  |
| Sachdeva 2024 [50] |  |  | X | X |  |  | X |  |  |  |  |  |  |  |  |  |  |  |
| Schmidt 2025 [51] |  | X | X |  |  |  | X |  | X |  |  |  |  |  |  |  |  |  |
| Shah 2022 [52] |  | X |  |  |  | X |  |  | X |  |  |  |  |  |  |  |  |  |
| Tirapelli 2025 [53] | X | X | X |  |  |  |  |  |  |  |  | X | X |  |  |  | X |  |
| Wahlich 2025 [54] | X |  |  | X | X |  |  |  |  |  | X | X |  | X |  |  |  |  |
| Whitestone 2024 [55] | X |  |  | X |  |  |  |  |  |  |  |  |  |  |  |  |  |  |
| Woode 2025 [56] | X |  |  | X |  | X |  |  |  |  |  |  | X |  |  |  |  |  |
| Xuereb 2024 [57] | X | X | X |  |  |  |  |  | X |  |  |  |  |  |  |  |  |  |
| Yakar 2022 [58] |  | X | X |  |  | X |  |  | X |  |  |  |  |  |  |  |  |  |
| Yap 2022 [59] | X | X |  |  |  |  |  | X |  |  |  |  |  |  |  |  |  |  |
| Ye 2019 [60] |  |  | X |  |  | X | X |  |  | X |  |  |  |  |  |  |  |  |
| York 2020 [61] |  | X | X |  |  |  |  |  | X |  |  |  |  |  |  |  |  |  |

# Results for aim (a): factors influencing patients’ perceptions of AI in medical image analysis

### Sociodemographic factors

Age

Overall, 33 studies reported association of patients’ age and their perception or acceptance of AI in medical image analysis. Across these studies, influence of age was inconsistent, and age was observed to have positive, negative, mixed, or no significant association with patient acceptance of AI.

No significant influence of age on AI acceptance was noted across 10/33 studies [7, 18, 24, 28, 29, 35, 36, 43, 44, 49]. Some studies reported that younger patients demonstrated greater acceptance of AI, which was potentially due to younger patients placing less importance on who their provider was (AI or human), their greater understanding of AI, and higher perceived value in AI and its potential [5, 9, 14, 19, 25, 26, 32, 52]. However, there was a report of younger participants not seeing the value in AI, this was in cases where the context was an uncurable disease [38]. In contrast, other studies noted that older participants expressed greater acceptance of AI, which was driven by factors such as their hope that AI might reduce waiting times or support early detection of diseases [6, 16, 21, 34, 47, 59]. However, older patients were also reported to lower appreciation and understanding of AI [8], hold higher skepticism [53] and lower trust or confidence in AI [12, 32, 41, 58, 61], and higher preference for their doctor’s involvement [57]. There was contrasting evidence with one study reporting greater acceptance of autonomous AI among older patients [51], while another reported greater resistance to autonomous systems among this group [47].

Gender

Thirteen studies reported whether gender influenced acceptance of AI in medical image analysis. Five of these studies reported no significant influence of gender on trust, confidence, or acceptance of AI [7, 13, 19, 26, 41]. Where gender-related differences were observed, male participants more frequently expressed higher acceptance of AI, greater trust in AI outputs, and a stronger belief in AI accuracy or its potential to replace clinicians [4, 51, 52, 58]. One study noted that male participants were more willing to wait longer to receive an additional computer-assisted diagnosis or input [16]. This study also observed a non-significant higher trust and perceived safety of an automated image analysis system among male patients. Whereas evidence indicated that women were more likely to believe the doctors to provide a reliable interpretation of their medical images over AI and placed more trust in radiologist-derived reports [57, 61]. Additionally, women reported greater concerns about the validity of AI outputs and its accountability [12].

Education and technological literacy

A total of 33 studies reported the influence of education level and technological literacy on patients’ acceptance of AI in medical image analysis. Most studies reported that higher education and greater technological literacy corresponded with greater acceptance of AI [5, 8, 9, 12, 18, 41, 43, 47, 51, 58]. Higher education also meant greater technological literacy which influenced patients’ trust in AI [4, 32]. Educated individuals showed more confidence in AI assessments, perceived it as safe, useful, and were more accepting of AI even as an autonomous tool [11, 14, 22, 24, 28, 38, 57]. Furthermore, familiarity with AI, higher eHealth Literacy Scale (eHEALS) scores, or frequent use of technology were associated with favourable views of AI and reduced apprehension [13, 14, 25, 49, 53, 61]. In one study, patients with lower education were found to be more likely to request human verification of AI decisions [42], while seven studies reported no significant association between education level or technological awareness and acceptance of AI [7, 26, 29, 37, 44, 49, 60]. There were also reports of a more complex pattern where those with higher education or technological awareness were more sceptical of AI, and questioned the credibility of AI and expressed concerns about potential malfunctions [21, 47, 50]. Some studies reported that participants with lower education or technological awareness expressed higher confidence in AI, because they trust the health care institution using it well or due to less concerns of technical limitations [50].

Other demographic and patient characteristics factors

Few studies reported on the association between socioeconomic status (SES) (n=2), geographic location (n=2), race/ethnicity (n=3), cultural factors (n=1) and acceptance of AI. Higher socioeconomic status or income were commonly associated with more favourable perceptions of AI, including greater preference for AI-assisted image analysis and reduced need for explicit permission prior to AI use [39, 43]. Patients’ geographic location also appeared to influence acceptance, with participants from urban settings expressing more optimistic views toward AI compared to those from rural backgrounds [5]. In one study, greater acceptance of AI replacing clinicians was observed in a specific urban region, potentially reflecting local health care burdens such as longer waiting times in the area [53]. Race and ethnicity were consistently linked to concerns about equity, bias, and trust. Participants from non-White or minority ethnic backgrounds were more likely to express concerns that AI systems may not perform equally across populations or may worsen existing biases in health care [14,43]. While some minority participants anticipated a greater role for AI in replacing clinicians, this expectation was often accompanied by a clear preference for continued human supervision [4]. Cultural considerations were explicitly addressed in only one qualitative study, which highlighted that AI technologies may conflict with patients’ culturally aligned ways of knowing, healing, or understanding health and illness [3]. Involving trusted community or cultural leaders in communication and decision-making processes was suggested to mitigate cultural barriers to AI adoption [3].

### Clinical experience and health factors

Factors associated with patients’ clinical experience and health factors were extracted from 20 studies. Four of these reported no significant influence of patients’ medical history and their acceptance of, or intention to use AI [16, 35, 43, 60].

Several studies however indicated that prior exposure to disease, screening, or diagnostic procedures increased acceptance of AI. Patients with a history of melanoma, who often underwent cancer screening, with urgent referrals, or prior diagnostic procedures (e.g. biopsy, X-ray) were more likely to express trust in AI, support its use, or accept greater autonomy for AI systems [4, 9, 23, 29, 30, 36, 45]. Similarly, patients with chronic conditions such as diabetes reported higher satisfaction with AI-based screening compared to those without the condition [52]. Prior negative clinical experiences, such as a missed fracture on X-ray were also associated with stronger beliefs that AI could outperform human interpretation [33].

Contrarily, patients with poorer perceived health status or higher subjective illness burden reported caution and reduced trust in AI [7, 24, 49]. One study further reported that in instances of a chronic or non-curable condition the enthusiasm for AI was lacking [38]. Three studies reported that those who were new to cancer screening processes or had not had a recent hospital encounter had fewer reservations and greater acceptance of AI [47, 56, 58]

### Psychosocial and ethical concerns

Privacy

Privacy related factors influencing acceptance of AI in medical image analysis were extracted from 12 of the included studies. Only one of these studies reported no influence of privacy factors on acceptance [26], whereas privacy concerns were consistently recorded as a barrier to the acceptance of AI across the other studies. Studies reported patients’ fears related to loss of privacy, data misuse, hacking, or unauthorized access to electronic medical records [10, 11, 14, 15, 43]. These concerns were amplified where patients perceived risk of commercial use of their data and poor data anonymization [20, 27, 29]. It was however noted that patients’ privacy concerns were largely mitigated by trust in their health care system [38]. Studies further emphasized that robust governance, adequate anonymization of data, and quality assurance mechanisms could offset privacy related anxieties and support acceptance [3, 54].

Trust

Influence of trust on patients’ acceptance of AI in medical image analysis were identified across multiple included studies (n=13) and consistently emerged as central to how patients evaluated and responded to AI-based interventions. Trust in AI was associated with greater acceptance of AI [10, 15, 34], and influenced how patients interpreted perceived usefulness of AI, which thereby influenced acceptance [60].

Trust extended beyond patients’ trust in the AI systems, with patients’ intentions to accept or use AI being influenced by the opinions of significant others, family members, peers, and leaders [60]. Additionally, trust in clinicians and health care institutions were noted to facilitate acceptance of AI [3, 10, 11, 14, 22, 27, 36, 38]. This was further reflected in evidence where patients who had direct communication with radiologists or whose clinicians recommended AI were more likely to view AI positively [14, 15]. This trust remained even in the absence of detailed technical understanding of AI [27]. Conversely, strong trust in clinicians’ capability and skills reduced acceptance of AI acting independently [46]. This further reinforces patient preferences for clinician-led or supervised AI deployment.

Acceptance was also noted to be high when AI was embedded within trusted, clinician-led workflows and when accountability and responsibility were clearly retained by human professionals. Furthermore, institutional ownership of AI systems also played a role, with greater trust demonstrated toward AI developed or governed by public or nationally affiliated health care organizations compared to commercial or foreign entities [45].

Fairness and bias

Fairness and bias related factors influencing acceptance were extracted from six of the included studies, with evidence indicating concerns of bias in AI could undermine patients trust and acceptance [14, 17, 45, 54]. These concerns were mainly influenced by patient characteristics such as their gender and race, with women worrying AI could broaden existing disparities and overlook preferences of women, and minorities being concerned regarding possible bias and AI failing to account for population variations [20, 43].

Accountability

Accountability factors were extracted from 10 studies. Clarity regarding responsibility for AI-related errors was reported as influencing patients’ acceptance of AI in medical image analysis [13, 18, 22, 47]. Concerns about responsibility, errors, and accountability negatively influenced acceptance [12]. Patients commonly expected that accountability should remain with humans rather than the AI system itself, reflecting the view of AI as a supportive tool rather than an autonomous decision maker [11, 27]. Clinicians, hospitals, and health care institutions were most frequently identified as the appropriate parties to hold responsibility for AI-related outcomes, followed by preferences for shared accountability across all involved stakeholders. In contrast, holding AI manufacturers or developers solely responsible was least preferred by patients [18, 43, 53, 56].

### Attributes of the AI-enabled service

Perceived benefits of AI

Patients perceived benefits of AI was reported to influence their acceptance of AI and was extracted from 13 studies. These perceived benefits included AI’s ability to improve efficiency, workflow, and quality of care [12, 15, 22, 50, 51]. The ability of AI to analyse large image datasets efficiently and consistently, and to potentially reduce human error, was viewed as a key advantage [10, 36]. Acceptance was further strengthened when AI was perceived as useful and safe [20, 21, 47]. Patients were more likely to accept AI when they believed it could improve diagnostic quality, enhance care delivery, enable earlier detection of disease, or prevent missed diagnoses, thereby improving clinical outcomes [38, 39, 60]. Where patients perceived AI as fallible, unsafe, or limited in managing novel or complex cases, the acceptance was lower [20, 46].

Role of AI and human-in-the-loop

The AI application approach and the degree of human involvement emerged as the most reported factor influencing patients’ acceptance of AI, being identified across the majority of the included studies (n = 47) and representing the highest extracted theme overall.

The autonomous use of AI for medical image analysis to support screening and diagnosis was the least preferred approach and was predominantly met with discomfort and rejection by patients [13, 18, 21, 25, 33, 48, 49, 57]. Human expertise was central to patients’ acceptance of the screening and diagnosis pathway [16, 20, 27, 34, 37, 41, 44, 48, 54], with acceptance being enhanced when AI was embedded within trusted clinician-led pathways [55]. Human interaction, both verbal communications, and non-verbal such as eye contact and empathy were highly valued [7, 9, 11, 15, 22, 40, 46]. It was noted that patients had a strong desire for human involvement in the review of their X-rays [33, 38], automated skin image analysis [19], mammogram interpretations [27, 42], and communication and discussion of results [9]. Concerns about over reliance on AI, AI making the final decision, or that the use of autonomous AI systems could diminish opportunities for discussion, depersonalise care negatively, or deskill clinicians were significant barriers to acceptance [10, 12, 24].

Patients believed that although AI may be a powerful tool it requires human oversight, and the greater control should lie with their health care professional to make the final decision [5, 17, 21, 27, 28, 57, 59]. Across studies, there was a strong preference among patients for augmented AI models, where AI is integrated into the diagnostic workflow to support, aid, or augment their health care providers decision and not replace them as an autonomous decision maker [4, 11, 17, 20, 24, 26, 27, 30, 32, 33, 38-40, 42, 44, 45, 47, 54, 56].

Additionally, patients preferred AI application approaches that were well integrated or aligned with existing clinical workflows, such as parallel readings by AI and clinicians or AI replacing one reader while maintaining human presence, as these models’ minimised disruption and preserved familiarity [19, 35, 56]. Additionally, portraying AI as a tool like system rather than a human like or autonomous system designed to replace clinicians improved acceptance [17, 36, 53].

Accuracy and performance

Accuracy of AI and its performance capability were reported as strong prerequisites for its acceptance [21, 40, 45, 54]. This influence was extracted from 23 of the studies. Patients placed high importance on receiving accurate results [9, 23], sometimes more than receiving a faster diagnosis [4, 33].  AI was often compared to clinicians, with patents requiring AI to perform better than humans to drive their acceptance [6, 10, 11, 13, 20, 27, 29].  A study reported that patients were willing to accept autonomous use of AI whose accuracy surpassed the radiologists [18]. Patients expected AI systems to undergo extensive evaluation, regular retraining, and continuous quality assurance prior to and during clinical use [3, 27]. Scientific evidence demonstrating safety, effectiveness, and real-world performance was viewed as essential [17, 22, 34, 38, 44]. Patients expressed expectations of very high or even perfect systems with 100% accuracy [20], particularly if AI were to function independently [48]. Despite the emphasis and importance placed on accuracy, patients stated that they would prefer the involvement and supervision of their health care provider to verify AI outputs irrespective of its proven accuracy [9, 33, 36, 46].

Transparency and patient autonomy

Transparency and procedural knowledge, including patients’ understanding of how AI is used and their ability to exercise choice emerged as a factor influencing acceptance of AI, and was reported across 25 studies.

A consistent pattern across studies were that greater transparency and patients’ understanding of AI increased their trust and acceptance [6, 15, 17, 36, 38, 54]. Studies that provided detailed technical background, educational materials, or clear explanations reported higher acceptance compared to similar interventions where such information was absent, suggesting that procedural knowledge acts as a facilitator [16, 44, 55, 56]. The explainability of AI and traceability of its decision making was considered essential to accept the intervention into the screening and diagnosis process [23]. The expectations ranged from having transparency of the roles and responsibility of AI, the information source used by AI to make judgements, clarity regarding how their data was being processed and used, and transparency of the systems performance, logic, and reasoning while interpreting their reports [3, 7, 9, 22, 40, 41]. Some patients perceived AI as an opportunity for greater transparency in their care, noting that AI generated reports were sometimes clearer or more informative than traditional clinician delivered interpretations [29, 32]. Additionally, patients expressed that they required their health care professionals and staff to be well equipped to explain the AI and its application to them [11, 44]. Contrarily, some patients across studies were not keen to receive detailed information about the AI intervention used in their medical image analysis [10, 38, 50]. They were more focused on receiving the screening or diagnosis and less concerned with how it was carried out [11].

Patient autonomy emerged as an important consideration with patients’ reporting being informed of AI use, having the right to choose AI, and following adequate consenting process was critical to their acceptance [15, 20, 24, 39, 43]. Acceptance was higher when patients perceived that their choice was preserved, including the option to request human only interpretation, and when AI use was clearly communicated prior to the procedure [39, 54].

### Operational factors of the health care system

Wait time

Waiting time and diagnostic speed were identified as factors influencing patients’ acceptance of AI in medical image analysis across 14 studies. A common expectation that patients had from the introduction of AI in their medical image analysis was an increased screening and diagnostic speed [3, 40] and reduced health care wait times [9, 10, 22, 29]. This was also a factor that attracted many patients to accept AI [11, 26, 31], as shorter wait times also meant early diagnosis and reduced anxiety among patients [9, 29]. Additionally, there were reports of patients willing to trade-off their health care professionals’ involvement and accept autonomous use of AI in their screening or diagnosis for shorter wait times [30, 59].

However, findings indicated that faster diagnosis was not universally prioritised. Across studies it was observed that patients were willing to wait longer when it ensured accuracy [33], or health care professional involvement [43].

Willingness to wait appeared to reflect practical considerations related to care delivery, rather than patients’ underlying preferences for AI or non-AI approaches [28]. Efficiency and timeliness can influence how patients weigh the benefits of AI, however it is not sufficient on its own to drive acceptance.

Cost

Cost considerations were identified as factors influencing patients’ acceptance of AI in medical image analysis across 12 studies. Several studies reported that cost was a salient concern and could hinder acceptance, particularly when AI was perceived as increasing health care expenses or worsening existing health care disparities [6, 11, 15, 29, 38, 53]. Lower perceived costs of AI supported patient acceptance of AI [13, 26], whereas some perceived that AI could help reduce health care costs [40]. Contrarily, there were also concerns that AI adoption motivated by cost-cutting rather than care improvement could negatively affect patient support, particularly within publicly funded health care systems [54]. Willingness to pay for AI-assisted diagnosis varied considerably. While many patients reported being willing to pay out of pocket, if necessary, particularly those with higher socioeconomic status [29, 39]. Others, including older adults and rural populations, were more reluctant to incur additional costs [5]. Findings indicated variability in how patients perceived the importance of cost, with its influence differing by context, health care system, and patient characteristics.

# Summary characteristics of included studies

**Table S3:** Summary of included studies

| **Study** | **Study type** | **Study Location** | **No. of partici-pants** | **Imaging context** | **AI context** | **JBI quality score** | **No. of factors** |
| --- | --- | --- | --- | --- | --- | --- | --- |
| Adams 2020 [3] | Qualitative (engagement workshop) | Canada | 17 | Radiology imaging | Hypothetical | 0.70 | 7 |
| Agarwal 2025 [4] | Quantitative - survey | United Kingdom | 146 | Radiology (Paediatric musculoskeletal radiographs) | Hypothetical | 1.00 | 6 |
| Arora 2025 [5] | Quantitative - survey | India | 1562 | Dental imaging | Hypothetical | 1.00 | 5 |
| Ayad 2023 [6] | Quantitative - survey | Germany | 265 | Dental imaging | Hypothetical | 1.00 | 4 |
| Baghdadi 2024 [7] | Quantitative - survey | Saudi Arabia | 382 | Radiology imaging | Hypothetical | 1.00 | 6 |
| Bahadir 2025 [8] | Quantitative - survey | Turkey | 272 | Dental imaging | Applied | 1.00 | 2 |
| Bahakeem 2023 [9] | Quantitative - survey | Saudi Arabia | 1024 | Radiology imaging | Hypothetical | 1.00 | 6 |
| Baillie 2025 [10] | Qualitative (semi-structured interviews) | United Kingdom | 9 | Cardiac echocardiography | Applied | 0.90 | 7 |
| Carter 2023 [11] | Mixed method – dialogue group + survey | Australia | 50 | Breast imaging | Hypothetical | 0.82 | 9 |
| Catalina 2023 [12] | Quantitative - survey | Spain | 379 | Radiology imaging | Hypothetical | 1.00 | 6 |
| Clements 2022 [13] | Quantitative - survey | Australia | 283 | Radiology imaging | Hypothetical | 0.71 | 6 |
| Dontchos 2025 [14] | Quantitative - survey | United States | 3532 | Breast imaging | Hypothetical | 1.00 | 6 |
| El-Sayed 2025 [15] | Quantitative - survey | UAE | 205 | Radiology imaging | Hypothetical | 0.71 | 6 |
| Fink 2018 [16] | Quantitative - survey | Germany | 65 | Dermatology imaging | Applied | 1.00 | 5 |
| Foresman 2025 [17] | Qualitative (focus group) | United States | 17 | Radiology imaging (CT scan of the spine/back) | Hypothetical | 0.9 | 4 |
| Fransen 2025 [18] | Quantitative - survey | Western Europe | 212 | Radiology (prostate cancer) | Hypothetical | 1.00 | 5 |
| Frühauf 2012 [19] | Quantitative - survey | Austria | 209 | Dermatology imaging | Applied | 0.86 | 3 |
| Gatting 2024 [20] | Qualitative (focus group) | United Kingdom | 64 | Breast imaging | Hypothetical | 1.00 | 6 |
| Goessinger 2024 [21] | Quantitative - survey | Switzerland | 205 | Dermatology imaging | Applied | 1.00 | 5 |
| Haan 2019 [22] | Qualitative (semi-structured interviews) | Netherlands | 20 | Radiology imaging (CT of chest and abdomen) | Hypothetical | 1.00 | 8 |
| Haggenmüller 2024 [23] | Quantitative - survey (discrete choice experiment) | Germany | 178 | Dermatology imaging | Hypothetical | 1.00 | 3 |
| Holen 2024 [24] | Quantitative - survey | Norway | 8355 | Breast imaging | Hypothetical | 0.71 | 5 |
| Ibba 2023 [25] | Quantitative - survey | Italy | 2119 | Radiology imaging | Hypothetical | 0.71 | 3 |
| Jagemann 2024 [26] | Quantitative - survey (choice-based conjoint) | Germany | 126 | Dermatology imaging | Hypothetical | 1.00 | 7 |
| Johansson 2024 [27] | Qualitative (semi-structured interviews) | Sweden | 16 | Breast imaging | Applied | 0.90 | 5 |
| Jonmarker 2019 [28] | Quantitative - survey | Sweden | 2196 | Breast imaging | Hypothetical | 0.71 | 4 |
| Jutzi 2020 [29] | Quantitative - survey | Germany | 298 | Dermatology imaging | Hypothetical | 0.71 | 9 |
| Kawsar 2023 [30] | Quantitative - survey | United Kingdom | 268 | Dermatology imaging | Hypothetical | 0.71 | 3 |
| Keel 2018 [31] | Quantitative survey (primarily survey based but 2 were interviewed) | Australia | 96 | Ophthalmology (diabetic retinopathy) | Applied | 0.75 | 1 |
| Kosan 2022 [32] | Quantitative - survey | Germany | 140 | Dental imaging | Hypothetical | 1.00 | 4 |
| Lee 2024 [33] | Quantitative - survey | United Kingdom | 171 | Radiology (pediatric bone x-ray) | Hypothetical | 0.71 | 4 |
| Lennox-Chhugani 2021 [34] | Mixed methods (Large-scale survey + qualitative focus groups) | United Kingdom | Survey: 4,096  Focus groups: 25 | Breast imaging | Hypothetical | 0.82 | 4 |
| Lim 2022 [35] | Quantitative - survey | United Kingdom | 603 | Dermatology imaging | Hypothetical | 0.71 | 3 |
| Lyso 2024 [36] | Qualitative (focus group) | Norway | 48 | Radiology (prostate MRI) | Hypothetical | 1.00 | 6 |
| Malerbi 2024 [37] | Quantitative - survey | Brazil | 121 | Ophthalmology (diabetic retinopathy) | Applied | 0.5 | 2 |
| Manning 2023 [38] | Qualitative (semi-structured focus groups) | United Kingdom | 14 | Radiology (Skeletal, Osteoporosis) | Hypothetical | 1.00 | 10 |
| McGhee 2025 [39] | Quantitative - survey | United states | 226 | Radiology imaging | Hypothetical | 0.86 | 5 |
| Nelson 2020 [40] | Qualitative (semi-structured interviews) | United Kingdom | 48 | Dermatology imaging | Hypothetical | 1.00 | 5 |
| Ongena 2020 [41] | Quantitative - Questionnaire development – survey | Netherlands | 155 | Radiology imaging | Hypothetical | 1.00 | 5 |
| Ongena 2021 [42] | Quantitative - survey | Netherlands | 922 | Breast imaging | Hypothetical | 1.00 | 2 |
| Ozcan 2025 [43] | Quantitative - survey | United States | 518 | Breast imaging | Hypothetical | 0.71 | 11 |
| Palmisciano 2020 [44] | Mixed method - survey | United Kingdom | Qualitative survey: 20 Quantitative survey: 107 | Brain imaging | Hypothetical | 0.65 | 5 |
| Pearce 2025 [45] | Quantitative - survey (discrete choice experiment) | Australia | 802 | Breast imaging | Hypothetical | 1.00 | 5 |
| Pelayo 2023 [46] | Qualitative (semi-structured interviews) | United states | 20 | Ophthalmology (diabetic retinopathy) | Hypothetical | 0.90 | 4 |
| Pesapane 2023 [47] | Quantitative - survey | Italy | 800 | Breast imaging | Hypothetical | 0.71 | 6 |
| Popic 2025 [48] | Qualitative (dialogue/focus groups) | Australia | 40 | Breast imaging | Hypothetical | 1.00 | 2 |
| Rodler 2024 [49] | Quantitative - survey | Germany | 466 | Radiology (Prostate MRI) | Hypothetical | 1.00 | 4 |
| Sachdeva 2024 [50] | Qualitative (semi-structured focus groups) | Cameroon | 32 | Cervical cancer | Hypothetical | 0.90 | 3 |
| Schmidt 2025 [51] | Quantitative - survey | United states | 508 | Endoscopic imaging (colonoscopy images) | Hypothetical | 0.86 | 4 |
| Shah 2022 [52] | Quantitative - survey | India | 104 | Ophthalmology (diabetic retinopathy) | Applied | 0.57 | 3 |
| Tirapelli 2025 [53] | Quantitative - survey | worldwide - 6 countries | 2581 | Dental imaging | Hypothetical | 0.86 | 6 |
| Wahlich 2025 [54] | Qualitative (free text survey) | United Kingdom | 387 | Ophthalmology (diabetic retinopathy) | Hypothetical | 0.90 | 6 |
| Whitestone 2024 [55] | Quantitative - survey | Sub-Saharan Africa | 823 | Ophthalmology (diabetic retinopathy) | Applied | 0.57 | 2 |
| Woode 2025 [56] | Quantitative - survey | Australia | 2063 | Breast imaging | Hypothetical | 1.00 | 4 |
| Xuereb 2024 [57] | Quantitative - survey | Malta | 280 | Radiology imaging | Hypothetical | 0.86 | 4 |
| Yakar 2022 [58] | Quantitative - survey | Netherlands | 1909 | Radiology and dermatology | Hypothetical | 1.00 | 4 |
| Yap 2022 [59] | Quantitative - survey | New Zealand | 438 | Ophthalmology (diabetic retinopathy) | Hypothetical | 0.71 | 3 |
| Ye 2019 [60] | Quantitative - survey | China | 474 | Ophthalmology (diabetic retinopathy) | Hypothetical | 1.00 | 4 |
| York 2020 [61] | Quantitative - survey | United Kingdom | 216 | Radiology (Skeletal) | Hypothetical | 0.57 | 3 |

# References

1. Page MJ, McKenzie JE, Bossuyt PM, Boutron I, Hoffmann TC, Mulrow CD, et al. The PRISMA 2020 statement: an updated guideline for reporting systematic reviews. bmj. 2021;372. doi:https://doi.org/10.1136/bmj.n71. PMID: 33782057
2. Joanna Briggs Institute. Critical Appraisal Tools. Critical Appraisal Tools [accessed 2025-08-31]. Available from: https://jbi.global/critical-appraisal-tools.
3. Adams SJ, Tang R, Babyn P. Patient Perspectives and Priorities Regarding Artificial Intelligence in Radiology: Opportunities for Patient-Centered Radiology. Journal of the American College of Radiology. 2020;17(8):1034-6. doi:10.1016/j.jacr.2020.01.007. PMID: 32068006
4. Agarwal G, Salami RK, Lee L, Martin H, Shantharam L, Thomas K, et al. Parental and carer views on the use of AI in imaging for children: a national survey. Insights into Imaging. 2025;16(1):172. doi:10.1186/s13244-025-02021-6. PMID: 40782183
5. Arora PC, Sandhu KK, Arora A, Gupta A, Waghmare M, Rampal V. Acceptability of artificial intelligence in dental radiology among patients in India: are we ready for this revolution? Oral Radiology. 2025;41(1):69-77. doi:10.1007/s11282-024-00777-z. PMID: 39384683
6. Ayad N, Schwendicke F, Krois J, van den Bosch S, Bergé S, Bohner L, et al. Patients' perspectives on the use of artificial intelligence in dentistry: a regional survey. Head Face Med. 2023;19(1):23. doi:10.1186/s13005-023-00368-z. PMID: 37349791
7. Baghdadi LR, Mobeirek AA, Alhudaithi DR, Albenmousa FA, Alhadlaq LS, Alaql MS, et al. Patients' Attitudes Toward the Use of Artificial Intelligence as a Diagnostic Tool in Radiology in Saudi Arabia: Cross-Sectional Study. JMIR Hum Factors. 2024;11:e53108. doi:https://doi.org/10.2196/53108. PMID: 39110973
8. Bahadir HS, Keskin NB, Çakmak EŞK, Güneç G, Cesur Aydin K, Peker F. Patients’ attitudes toward artificial intelligence in dentistry and their trust in dentists. Oral Radiology. 2025;41(1):52-9. doi:10.1007/s11282-024-00775-1. PMID: 39379636
9. Bahakeem BH, Alobaidi SF, Alzahrani AS, Alhasawi R, Alzahrani A, Alqahtani W, et al. The General Population's Perspectives on Implementation of Artificial Intelligence in Radiology in the Western Region of Saudi Arabia. Cureus. 2023;15(4):e37391. doi:https://doi.org/10.7759/cureus.37391. PMID: 37182053
10. Baillie L, Stewart-Lord A, Thomas N, Frings D. Patients’, clinicians’ and developers’ perspectives and experiences of artificial intelligence in cardiac healthcare: A qualitative study. DIGITAL HEALTH. 2025;11:20552076251328578. doi:10.1177/20552076251328578. PMID: 40534891
11. Carter SM, Carolan L, Saint James Aquino Y, Frazer H, Rogers WA, Hall J, et al. Australian women's judgements about using artificial intelligence to read mammograms in breast cancer screening. Digital Health. 2023;9. doi:https://doi.org/10.1177/20552076231191057. PMID: 37559826
12. Miró Catalina Q, Femenia J, Fuster-Casanovas A, Marin-Gomez FX, Escalé-Besa A, Solé-Casals J, et al. Knowledge and Perception of the Use of AI and its Implementation in the Field of Radiology: Cross-Sectional Study. Journal of medical Internet research. 2023;25:e50728. doi:https://doi.org/10.2196/50728. PMID: 37831495
13. Clements W, Thong LP, Zia A, Moriarty HK, Goh GS. A Prospective Study Assessing Patient Perception of the Use Of Artificial Intelligence in Radiology. Asia Pacific Journal of Health Management. 2022;17(1). doi:https://doi.org/10.24083/apjhm.v17i1.861. Corpus ID: 248054054
14. Dontchos BN, Dodelzon K, Bhole S, Edmonds CE, Mullen LA, Parikh JR, et al. Opinions and Preferences Regarding Artificial Intelligence Use in Health Care Delivery: Results From a National Multisite Survey of Breast Imaging Patients. Journal of the American College of Radiology. 2025;22(9):1032-40. doi:10.1016/j.jacr.2025.05.001. PMID: 40339678
15. El-Sayed MZ, Rawashdeh M, Moossa A, Atfah M, Prajna B, Ali MA. Patient perspectives on AI in radiology: Insights from the United Arab Emirates. Clinical Imaging. 2025;125. doi:10.1016/j.clinimag.2025.110543. PMID: 40513450
16. Fink C, Uhlmann L, Hofmann M, Forschner A, Eigentler T, Garbe C, et al. Patient acceptance and trust in automated computer-assisted diagnosis of melanoma with dermatofluoroscopy. J Dtsch Dermatol Ges. 2018;16(7):854-9. doi:10.1111/ddg.13562. PMID: 29927518
17. Foresman G, Biro J, Tran A, MacRae K, Kazi S, Schubel L, et al. Patient Perspectives on Artificial Intelligence in Health Care: Focus Group Study for Diagnostic Communication and Tool Implementation. J Particip Med. 2025;17:e69564. doi:10.2196/69564. PMID: 40705399
18. Fransen SJ, Kwee TC, Rouw D, Roest C, van Lohuizen QY, Simonis FFJ, et al. Patient perspectives on the use of artificial intelligence in prostate cancer diagnosis on MRI. European Radiology. 2025;35(2):769-75. doi:10.1007/s00330-024-11012-y. PMID: 39143247
19. Frühauf J, Leinweber B, Fink-Puches R, Ahlgrimm-Siess V, Richtig E, Wolf IH, et al. Patient acceptance and diagnostic utility of automated digital image analysis of pigmented skin lesions. Journal of the European Academy of Dermatology and Venereology. 2012;26(3). doi:https://doi.org/10.1111/j.1468-3083.2011.04081.x. PMID: 21504486
20. Gatting L, Ahmed S, Meccheri P, Newlands R, Kehagia AA, Waller J. Acceptability of artificial intelligence in breast screening: focus groups with the screening-eligible population in England. BMJ Public Health. 2024;2(2):e000892. doi:10.1136/bmjph-2024-000892. PMID: 40018529
21. Goessinger EV, Niederfeilner JC, Cerminara S, Maul JT, Kostner L, Kunz M, et al. Patient and dermatologists' perspectives on augmented intelligence for melanoma screening: A prospective study. Journal of the European Academy of Dermatology and Venereology. 2024. doi:10.1111/jdv.19905. PMID: 38411348
22. Haan M, Ongena YP, Hommes S, Kwee TC, Yakar D. A Qualitative Study to Understand Patient Perspective on the Use of Artificial Intelligence in Radiology. Journal of the American College of Radiology. 2019;16(10):1416-9. doi:10.1016/j.jacr.2018.12.043. PMID: 30878311
23. Haggenmüller S, Maron RC, Hekler A, Krieghoff-Henning E, Utikal JS, Gaiser M, et al. Patients' and dermatologists' preferences in artificial intelligence-driven skin cancer diagnostics: A prospective multicentric survey study. J Am Acad Dermatol. 2024;91(2):366-70. doi:10.1016/j.jaad.2024.04.033. PMID: 38670313
24. Sørlien Holen Å, Almenning Martiniussen M, Burns Bergan M, Moshina N, Hovda T, Hofvind S. Women's attitudes and perspectives on the use of artificial intelligence in the assessment of screening mammograms. European Journal of Radiology. 2024;175. doi:10.1016/j.ejrad.2024.111431. PMID: 38520804
25. Ibba S, Tancredi C, Fantesini A, Cellina M, Presta R, Montanari R, et al. How do patients perceive the AI-radiologists interaction? Results of a survey on 2119 responders. European Journal of Radiology. 2023;165. doi:https://doi.org/10.1016/j.ejrad.2023.110917. PMID: 37327548
26. Jagemann I, Wensing O, Stegemann M, Hirschfeld G. Acceptance of Medical Artificial Intelligence in Skin Cancer Screening: Choice-Based Conjoint Survey. JMIR Formative Research. 2024;8. doi:https://doi.org/10.2196/46402. PMID: 38214959
27. Viberg Johansson J, Dembrower K, Strand F, Grauman Å. Women's perceptions and attitudes towards the use of AI in mammography in Sweden: a qualitative interview study. BMJ Open. 2024;14(2):e084014. doi:10.1136/bmjopen-2024-084014. PMID: 38355190
28. Jonmarker O, Strand F, Brandberg Y, Lindholm P. The future of breast cancer screening: what do participants in a breast cancer screening program think about automation using artificial intelligence? Acta Radiol Open. 2019;8(12):2058460119880315. doi:https://doi.org/10.1177/2058460119880315. PMID: 31839989
29. Jutzi TB, Krieghoff-Henning EI, Holland-Letz T, Utikal JS, Hauschild A, Schadendorf D, et al. Artificial Intelligence in Skin Cancer Diagnostics: The Patients' Perspective. Frontiers in Medicine. 2020;7. doi:https://doi.org/10.3389/fmed.2020.00233. PMID: 32671078
30. Kawsar A, Hussain K, Kalsi D, Kemos P, Marsden H, Thomas L. Patient perspectives of artificial intelligence as a medical device in a skin cancer pathway. Frontiers in Medicine. 2023;10. doi:https://doi.org/10.3389/fmed.2023.1259595. PMID: 38046409
31. Keel S, Lee PY, Scheetz J, Li Z, Kotowicz MA, MacIsaac RJ, et al. Feasibility and patient acceptability of a novel artificial intelligence-based screening model for diabetic retinopathy at endocrinology outpatient services: A pilot study. Scientific Reports. 2018;8(1). doi:10.1038/s41598-018-22612-2. PMID: 29531299. PMID: 29531299
32. Kosan E, Krois J, Wingenfeld K, Deuter CE, Gaudin R, Schwendicke F. Patients' Perspectives on Artificial Intelligence in Dentistry: A Controlled Study. J Clin Med. 2022;11(8). doi:10.3390/jcm11082143. PMID: 35456236
33. Lee L, Salami RK, Martin H, Shantharam L, Thomas K, Ashworth E, et al. "How I would like AI used for my imaging": children and young persons' perspectives. Eur Radiol. 2024. doi:10.1007/s00330-024-10839-9. PMID: 38900281
34. Lennox-Chhugani N, Chen Y, Pearson V, Trzcinski B, James J. Women's attitudes to the use of AI image readers: A case study from a national breast screening programme. BMJ Health and Care Informatics. 2021;28(1). doi:https://doi.org/10.1136/bmjhci-2020-100293. PMID: 33795236
35. Lim K, Neal-Smith G, Mitchell C, Xerri J, Chuanromanee P. Perceptions of the use of artificial intelligence in the diagnosis of skin cancer: an outpatient survey. Clinical and Experimental Dermatology. 2022;47(3). doi:https://doi.org/10.1111/ced.14969. PMID: 34610153
36. Lysø EH, Hesjedal MB, Skolbekken JA, Solbjør M. Men's sociotechnical imaginaries of artificial intelligence for prostate cancer diagnostics – A focus group study. Social Science and Medicine. 2024;347. doi:10.1016/j.socscimed.2024.116771. PMID: 38537333
37. Malerbi FK, Mezzomo Ventura B, Fischer M, Penha FM. Patients Perceptions of Artificial Intelligence in a Deep Learning-Assisted Diabetic Retinopathy Screening Event: A Real-World Assessment. J Diabetes Sci Technol. 2024;18(3):750-1. doi:https://doi.org/10.1177/19322968241234378. PMID: 38404014
38. Manning F, Mahmoud A, Meertens R. Understanding patient views and acceptability of predictive software in osteoporosis identification. Radiography. 2023;29(6):1046-53. doi:https://doi.org/10.1016/j.radi.2023.08.011. PMID: 37734275
39. McGhee KN, Barrett DJ, Safarini O, Elkassem AA, Eddins JT, Smith AD, et al. Patient Preferences for Artificial Intelligence in Medical Imaging: A Single-Center Cross-Sectional Survey. Journal of Imaging Informatics in Medicine. 2025. doi:10.1007/s10278-025-01629-w. PMID: 40775178
40. Nelson CA, Pérez-Chada LM, Creadore A, Li SJ, Lo K, Manjaly P, et al. Patient Perspectives on the Use of Artificial Intelligence for Skin Cancer Screening: A Qualitative Study. JAMA Dermatology. 2020;156(5). doi:https://doi.org/10.1001/jamadermatol.2019.5014. PMID: 32159733
41. Ongena YP, Haan M, Yakar D, Kwee TC. Patients' views on the implementation of artificial intelligence in radiology: development and validation of a standardized questionnaire. Eur Radiol. 2020;30(2):1033-40. doi:10.1007/s00330-019-06486-0. PMID: 31705254
42. Ongena YP, Yakar D, Haan M, Kwee TC. Artificial Intelligence in Screening Mammography: A Population Survey of Women's Preferences. Journal of the American College of Radiology. 2021;18(1):79-86. doi:https://doi.org/10.1016/j.jacr.2020.09.042. PMID: 33058789
43. Ozcan BB, Dogan BE, Xi Y, Knippa EE. Patient Perception of Artificial Intelligence Use in Interpretation of Screening Mammograms: A Survey Study. Radiology: Imaging Cancer. 2025;7(3):e240290. doi:10.1148/rycan.240290. PMID: 40249272
44. Palmisciano P, Jamjoom AAB, Taylor D, Stoyanov D, Marcus HJ. Attitudes of Patients and Their Relatives Toward Artificial Intelligence in Neurosurgery. World Neurosurgery. 2020;138:e627-e33. doi:https://doi.org/10.1016/j.wneu.2020.03.029. PMID: 32179185
45. Pearce A, Carter S, Frazer HM, Houssami N, Macheras-Magias M, Webb G, et al. Implementing artificial intelligence in breast cancer screening: Women's preferences. Cancer. 2025;131(9):e35859. doi:10.1002/cncr.35859. PMID: 40262029
46. Pelayo C, Hoang J, Pinzón MM, Lock LJ, Fowlkes C, Stevens CL, et al., editors. Perspectives of Latinx Patients with Diabetes on Teleophthalmology, Artificial Intelligence-Based Image Interpretation, and Virtual Care: A Qualitative Study. Telemedicine Reports; 2023. PMID: 37908628
47. Pesapane F, Rotili A, Valconi E, Agazzi GM, Montesano M, Penco S, et al. Women's perceptions and attitudes to the use of AI in breast cancer screening: a survey in a cancer referral centre. Br J Radiol. 2023;96(1141):20220569. doi:10.1259/bjr.20220569. PMID: 36314388
48. Popic D, Marinovich ML, Houssami N, Hall J, Carter SM. How should artificial intelligence be used in breast screening? Women’s reasoning about workflow options. PLOS ONE. 2025;20(5):e0323528. doi:10.1371/journal.pone.0323528. PMID: 40446203
49. Rodler S, Kopliku R, Ulrich D, Kaltenhauser A, Casuscelli J, Eismann L, et al. Patients' Trust in Artificial Intelligence-based Decision-making for Localized Prostate Cancer: Results from a Prospective Trial. Eur Urol Focus. 2023. doi:10.1016/j.euf.2023.10.020. PMID: 37923632
50. Sachdeva M, Datchoua AM, Yakam VF, Kenfack B, Jonnalagedda-Cattin M, Thiran JP, et al. Acceptability of artificial intelligence for cervical cancer screening in Dschang, Cameroon: a qualitative study on patient perspectives. Reproductive Health. 2024;21(1). doi:https://doi.org/10.1186/s12978-024-01828-8. PMID: 38937771
51. Schmidt KA, Sood S, Dilmaghani S, Leggett C, Dierkhising R, Goyal M, et al. Understanding Patients’ Current Acceptability of Artificial Intelligence During Colonoscopy for Polyp Detection: A Single-Center Study. Techniques and Innovations in Gastrointestinal Endoscopy. 2025;27(2). doi:10.1016/j.tige.2024.250905
52. Shah P, Mishra D, Shanmugam M, Vighnesh MJ, Jayaraj H. Acceptability of artificial intelligence-based retina screening in general population. Indian Journal of Ophthalmology. 2022;70(4):1140-4. doi:https://doi.org/10.4103/ijo.IJO_1840_21. PMID: 35326001
53. Tirapelli C, Gaêta-Araujo H, Costa ED, Scarfe WC, Oliveira-Santos C, Fischer KM, et al. Patient perceptions of artificial intelligence in dental imaging diagnostics: a multicentre survey. Dentomaxillofacial Radiology. 2025;54(6):427-36. doi:10.1093/dmfr/twaf018. PMID: 40080713
54. Wahlich C, Chandrasekaran L, Chaudhry UAR, Willis K, Chambers R, Bolter L, et al. Patient and practitioner perceptions around use of artificial intelligence within the English NHS diabetic eye screening programme. Diabetes Research and Clinical Practice. 2025;219. doi:10.1016/j.diabres.2024.111964. PMID: 39709112
55. Whitestone N, Nkurikiye J, Patnaik JL, Jaccard N, Lanouette G, Cherwek DH, et al. Feasibility and acceptance of artificial intelligence-based diabetic retinopathy screening in Rwanda. British Journal of Ophthalmology. 2024;108(6):840-5. doi:10.1136/bjo-2022-322683. PMID: 37541766
56. Woode ME, De Silva Perera U, Degeling C, Aquino YSJ, Houssami N, Carter SM, et al. Preferences for the Use of Artificial Intelligence for Breast Cancer Screening in Australia: A Discrete Choice Experiment. The Patient - Patient-Centered Outcomes Research. 2025;18(5):495-510. doi:10.1007/s40271-025-00742-w. PMID: 40347323
57. Xuereb F, Portelli DJL. The knowledge and perception of patients in Malta towards artificial intelligence in medical imaging. J Med Imaging Radiat Sci. 2024;55(4):101743. doi:https://doi.org/10.1016/j.jmir.2024.101743. PMID: 39317135
58. Yakar D, Ongena YP, Kwee TC, Haan M. Do People Favor Artificial Intelligence Over Physicians? A Survey Among the General Population and Their View on Artificial Intelligence in Medicine. Value in health : the journal of the International Society for Pharmacoeconomics and Outcomes Research. 2022;25(3):374-81. doi:https://doi.org/10.1016/j.jval.2021.09.004. PMID: 35227448
59. Yap A, Wilkinson B, Chen E, Han L, Vaghefi E, Galloway C, et al. Patients Perceptions of Artificial Intelligence in Diabetic Eye Screening. Asia-Pacific Journal of Ophthalmology. 2022;11(3):287-93. doi:https://doi.org/10.1097/APO.0000000000000525. PMID: 35772087
60. Ye T, Xue J, He M, Gu J, Lin H, Xu B, et al. Psychosocial factors affecting artificial intelligence adoption in health care in China: Cross-sectional study. Journal of Medical Internet Research. 2019;21(10). doi:https://doi.org/10.2196/14316. PMID: 31625950
61. York T, Jenney H, Jones G. Clinician and computer: a study on patient perceptions of artificial intelligence in skeletal radiography. BMJ Health & Care Informatics. 2020;27(3):e100233. doi:https://doi.org/10.1136/bmjhci-2020-100233. PMID: 33187956
